# Release of transcriptional repression through the HCR promoter region confers uniform expression of *HWP1* on surfaces of *Candida albicans* germ tubes

**DOI:** 10.1371/journal.pone.0192260

**Published:** 2018-02-13

**Authors:** Samin Kim, Quoc Bao Nguyen, Michael J. Wolyniak, Gregory Frechette, Christian R. Lehman, Brandon K. Fox, Paula Sundstrom

**Affiliations:** 1 Department of Microbiology and Immunology, Microbiology and Molecular Pathogenesis Program, Geisel School of Medicine at Dartmouth, Hanover, New Hampshire, United States of America; 2 Department of Biology, Hampden-Sydney College, Hampden-Sydney, Virginia, United States of America; King's College London Dental Institute, UNITED KINGDOM

## Abstract

The mechanisms that fungi use to co-regulate subsets of genes specifically associated with morphogenic states represent a basic unsolved problem in fungal biology. *Candida albicans* is an important model of fungal differentiation both for rapid interconversion between yeast and hyphal growth forms and for white/opaque switching mechanisms. The Sundstrom lab is interested in mechanisms regulating hypha-specific expression of adhesin genes that are critical for *C*. *albicans* hyphal growth phenotypes and pathogenicity. Early studies on hypha-specific genes such as *HWP1* and *ALS3* reported 5’ intergenic regions that are larger than those typically found in an average promoter and are associated with hypha-specific expression. In the case of *HWP1*, activation and repression involves a 368 bp region, denoted the *HWP1* control region (HCR), located 1410 bp upstream of its transcription start site. In previous work we showed that HCR confers developmental regulation to a heterologous *ENO1* promoter, indicating that HCR by itself contains sufficient information to couple gene expression to morphology. Here we show that the activation and repression mediated by HCR are localized to distinct HCR regions that are targeted by the transcription factors Nrg1p and Efg1p. The finding that Efg1p mediates both repression via HCR under yeast morphological conditions and activation conditions positions Efg1p as playing a central role in coupling *HWP1* expression to morphogenesis through the HCR region. These localization studies revealed that the 120 terminal base pairs of HCR confer Efg1p-dependent repressive activity in addition to the Nrg1p repressive activity mediated by DNA upstream of this subregion. The 120 terminal base pair subregion of HCR also contained an initiation site for an *HWP1* transcript that is specific to yeast growth conditions (HCR-Y) and may function in the repression of downstream DNA. The detection of an *HWP1* mRNA isoform specific to hyphal growth conditions (HWP1-H) showed that morphology-specific mRNA isoforms occur under both yeast and hyphal growth conditions. Similar results were found at the *ALS3* locus. Taken together, these results, suggest that the long 5’ intergenic regions upstream of hypha-specific genes function in generating mRNA isoforms that are important for morphology-specific gene expression. Additional complexity in the *HWP1* promoter involving HCR-independent activation was discovered by creating a strain lacking HCR that exhibited variable *HWP1* expression during hyphal growth conditions. These results show that while HCR is important for ensuring uniform *HWP1* expression in cell populations, HCR independent expression also exists. Overall, these results elucidate HCR-dependent mechanisms for coupling *HWP1*-dependent gene expression to morphology uniformly in cell populations and prompt the hypothesis that mRNA isoforms may play a role in coupling gene expression to morphology in *C*. *albicans*.

## Introduction

Morphotype-associated gene expression is a hallmark of fungi in general and is critical for virulence for a number of dimorphic fungal pathogens where growth in the host is constrained to either a yeast or specialized growth form [1]. For other fungi exhibiting both yeast and hyphal growth in host tissue, such as *Candida albicans*, reversible yeast/hypha transitions are important for several pathogenic processes such as adherence and biofilm formation via morphology-coupled gene expression. Morphology-associated cell surface changes are notable for helping fungi to defend against immune attack [1], and co-expression of morphology-associated cell surface proteins may lead to synergy between gene products for establishing biofilm formation and growth in the host. Consequently, interference *with C*. *albicans'* ability to couple gene expression to changes in morphology may potentially lead to therapeutic strategies for inhibiting fungal pathogen survival in host tissues [2].

*C*. *albicans* is estimated to cause over 400,000 life-threatening infections per year worldwide, with most infections occurring in economically-developed regions [3]. Host epithelial surfaces, which form the natural ecological niche for this opportunistic pathogen, may become heavily colonized by *C*. *albicans* as a result of a variety of risk factors, including HIV, immunosuppression, trauma, low birth weight, inhaling corticosteroids, and treatment with antibacterial drugs [4]. Leading risk factors for life-threatening disseminated candidiasis include the presence of a central venous catheter and colonization of mucosal surfaces [5]. Both of these risk factors may involve biofilms that form on abiotic catheter material on host surfaces and are dependent on morphotype-associated gene expression [6, 7].

How hypha-specific gene expression is coupled to morphology is a process that is incompletely understood. Early research to understand the connections between morphology and gene expression in *C*. *albicans* led to the discovery of *HWP1* and *ALS3*, two genes that are highly upregulated under hyphal growth conditions. The transcription factors Efg1p and Nrg1p were subsequently found to be important regulators of *HWP1*, *ALS3*, and other hypha-specific genes [8, 9]. Hypha-specific genes in *C*. *albicans* are controlled by non-canonical mechanisms and involve long (>2 kb) upstream intergenic regions. Distal activating regions 1.5 kb upstream of *HWP1* and *ALS3* [10, 11] undergo chromatin changes during induction [12, 13] in a manner reminiscent of developmentally-regulated genes in higher eukaryotes [14]. Accumulating evidence suggests that these long upstream regions are correlated with growth phase-specific expression by facilitating the expression of morphology-specific mRNA isoforms. In pioneering work on Efg1p, 5 ‘UTRs of different lengths in opaque and white growth phases of *C*. *albicans* were found [15], heralding the discovery of mRNA isoforms in opaque and white growth forms for other genes. Additional genes with 5’ UTRs of different lengths in opaque and white growth phases were identified in transcriptome analyses of the *C*. *albicans* genome, and these extended 5’ UTRs were predicted to have regulatory properties that mediated cell type-specific expression [16]. While transcription factors such as Efg1p and Nrg1p have been shown to be generally important for activation and repression of hypha-specific genes [12, 13], more research is needed to understand how these activation and repression events are coupled to the genetic program governing morphogenesis in the context of long 5' UTRs.

In our efforts to understand how *C*. *albicans* couples transcriptional programs to morphogenesis, we have focused on *HWP1* and identified a 368 bp region termed HCR (Hwp1 Control Region) within the 2 kb upstream promoter region that confers hypha-specific expression to a reporter gene, albeit to only half the level of activation of the complete upstream region [11]. Blast analyses of the *C*. *albicans* genome did not identify sequences related to HCR, however an HCR-like sequence was identified upstream of the *HWP1* gene in *C*. *dubliniensis*. The function of HCR may require specific architectural features that are required for morphology specific gene expression. To gain insight into HCR function, we mapped HCR sub-regions for their role in activating or repressing expression in hypha and yeast growth conditions and investigated the effect of HCR deletion on Hwp1p expression. Here, we show that activation and repression via HCR are localized to distinct HCR subregions targeted by the transcription factors Nrg1p and Efg1p. The identification of a repressor region distal to the putative Nrg1p binding site suggests that mechanisms other than loss of Nrg1p binding may be important for HCR repression under yeast growth conditions. Further analyses led to the discovery of a transcript initiating within the repressor subregion, suggesting the possibility that distinct mRNA isoforms play a role in the repression of *HWP1* during yeast growth conditions. This finding is similar to one that we and others have made in identifying a yeast condition-specific mRNA isoform of *ALS3* as well as hypha condition-specific mRNA isoforms initiating upstream of both the *HWP1* and *ALS3* coding regions. Together, these results shed new insights in to the complexity governing hypha-specific genes in *C*. *albicans* and prompt testable hypotheses about the mechanisms that couple *HWP1* gene expression to filamentous growth.

## Materials and methods

### Strains and growth conditions

A complete list of strains is shown in S1 Table. *C*. *albicans* strains were stored at -80°C and plated on yeast peptone dextrose (YPD) plates prior to use. Yeast strains grown for 48 h to stationary phase in liquid yeast nutrient broth (YNB) medium at 30°C and 250 rpm shaking were washed in PBS and inoculated at a concentration of 5x10^6^ yeast/mL into Medium 199 (ThermoFisher Scientific, Grand Island, NY) for germ tube formation (hyphal growth) or YNB for yeast growth. In both cases, cells were incubated at 37°C for 2.5 h at 100 rpm unless otherwise indicated. Other conditions for germ tube formation included Medium 199 in an atmosphere of 5% CO_2_, YPD with 10% bovine calf serum [17], modified Lee’s medium, pH 6.8 [18], or NAG medium containing N-acetylglucosamine (5 mM) (Sigma-Aldrich, St. Louis, MO), glucose (0.2%), PBS pH 7.0 (25 mM), and YNB salts (0.17%).

To assess the importance of HCR in physiological conditions, unstimulated whole human saliva was collected in sterile tubes kept on ice and clarified by centrifugation at 10,000 x g at 4°C for 30 m. The supernatant was used undiluted within 2 to 3 h after collection [19]. Cultures were examined microscopically to verify that greater than 95% of the cells formed germ tubes in each inducing medium.

The GFP reporter strains used to determine the role of HCR under different growth conditions were strains S and -1902 [11]. Strains MMC3 [20] and HLC52 [21] were the parental strains for determining the roles of Nrg1p and Efg1p, respectively, in HCR-mediated expression. Strain HCRENO [11] and other identical, independently-created strains were used to quantitate the expression of HCR. Strains used for other experiments are described below and in S1 Table.

Luria broth (LB) or agar was used for the growth of *Escherichia coli* strains. TOP10 (Invitrogen, Carlsbad, CA) competent cells used for cloning were grown in the presence of LB plus 100 μg/mL ampicillin, 25 μg/mL kanamycin, or 50 μg/mL zeomycin for plasmid selection and maintenance.

### Biofilm formation

*C*. *albicans* biofilms were grown according to previously-described methods [22] except that relative fluorescence was used to measure fungal growth. *C*. *albicans* strains were grown in YPD medium for 24 h at 27°C with constant 250 rpm rotation. Cells were harvested by centrifugation, resuspended in PBS, and enumerated in a hemocytometer. Based on these counts, the cells were suspended in Medium 199 at a concentration of 1x10^6^/mL and dispensed into a 96 well plate (0.1 mL per well) in quadruplicate. Plates were incubated with 100 rpm rotation for 90 min at 30°C prior to removal of supernatant and rinsed with 0.1 mL PBS prior to addition of 0.1 mL fresh Medium 199 per well. Plates were then incubated with 100 rpm rotation overnight at 37°C. Medium 199 was removed and wells were washed with PBS followed by the addition of 0.1 mL PBS prior to determining relative fluorescence by fluorometry as previously described [11].

### Monitoring GFP levels in reporter constructs by fluorometry

Fluorometry was carried out as previously described [11]. Briefly, cells from 5 mL of culture were washed twice with ice-cold phosphate-buffered saline (PBS), resuspended in 0.1 mL PBS, and transferred into a 96-well Cytoplate (ThermoFisher). GFP fluorescence was measured using a Victor2 1420 multilabel counter (Perkin-Elmer, Waltham, MA) and normalized by dividing the fluorescence emitted per 1 s exposure by the absorbance recorded at a wavelength of 600 nm to produce relative fluorescence units.

### Strain construction

#### Generation of strains for mapping subregions of HCR for function

To create strains that would test the role of HCR and its subregions in gene activation and repression, HCR or HCR sub-regions with XhoI and EcoRV restriction sites added were PCR amplified using *C*. *albicans* strain SC5314 genomic DNA as the template followed by digestion with XhoI and EcoRV. The fragments were gel purified and ligated into the pEX-1 plasmid backbone [11]. The following recombinant plasmids were subsequently created: pSKD77 (HCR, -1410 to -1042), pHCRa (HCRa region, -1410 to -1162), pHCRb (HCRb region, -1410 to -1235), pSKD78 (HCRc region, -1367 to -1042) and pSKD76 (HCRd region, -1367 to -1162). All listed positions correspond to the transcriptional start site of *HWP1* [23].

pSKD77, pSKD76 and pSKD78 were each transformed into *C*. *albicans* strain CAI4 to create strains HCR (SKD231), HCRd (SKD232) and HCRc (SKD233), respectively. Similarly, plasmids pHCRa and pHCRb were transformed into strain CAI4 to create strains HCRa and HCRb, respectively. All plasmids were inserted at the *ENO1* locus as previously described [11].

#### Generation of strains for studying the role of HCR and HCRd in *nrg1* and *efg1* mutants

Plasmids pSKD599 and pSKD600, which are identical to plasmids pSKD76 (HCRd) and pSKD77 (HCR), respectively, except for the substitution of *URA3* with NAT^R^ as the selectable marker, were transformed into *efg1∆/efg1∆* (HLC52) [21] and *nrg1∆/nrg1∆* (MMC3) [20] backgrounds, respectively, and integrated at the *ENO1* locus. This resulted in the generation of strains SKD501 (HCRd) and SKD502 (HCR) in the MMC3 background and SKD503 (HCRd) and SKD504 (HCR) in the HLC52 background. Control strains were created by transforming plasmids pSKD599 and pSKD600 into CAI4 to create strains SKD245 (HCRd) and SKD246 (HCR).

#### Generation of a strain with HCR deleted upstream of *HWP1*

Two PCR amplified fragments flanking HCR, –1641 to –1410 (with KpnI and ApaI sites added) and –1042 to –913 (with SacI and NotI sites added), were cloned into pJK863 [24] and pSFU [25, 26], which contained an FLP-recyclable *C*. *albicans NAT1* gene and *URA3* gene, respectively, surrounded by upstream KpnI and ApaI and downstream SacI and NotI sites. The PCR fragments and plasmid backbones were digested with KpnI-ApaI and SacI-NotI and gel purified prior to ligating the PCR amplified fragments on either side of the flipper cassette to create plasmids pSKD592 and pSKD593.

pSKD593 was integrated upstream of *HWP1* by homologous recombination in strain CAI4 to create SKD1 (HCR/∆*hcr*::NAT^R^). Subsequently, pSKD592 was transformed into SKD1 to create SKD3 (hcr∆::*URA3/*hcr∆::*NAT*^*R*^), a strain that is selectable on a YNB plate supplemented with nourseothricin and lacking uridine. Cells from strain SKD3 were then grown in YCB-BSA to allow for FLP-mediated excision of the *SAT1* flipper cassette, thereby creating *C*. *albicans* strain SKD4 (hcr∆/hcr∆).

#### Generation of a strain deleted for the entire *HWP1* locus

A flipper cassette strategy was used to delete the *HWP1* gene and its upstream and downstream regions [24]. Two fragments, one flanking the upstream (–2450 to –1917) and the other the downstream (+2633 to +3108) *HWP1* regions were PCR amplified from the genome with KpnI and ApaI (upstream fragment) or SacI and NotI (downstream fragment) sites added. The two fragments were digested with KpnI-ApaI and SacI-NotI, respectively, and ligated into pJK863, which was also digested with KpnI-ApaI and SacI-NotI on either side of the flipper cassette, creating pSKD541.

Recombinant plasmid pSKD541 was used to transform CAI4 to generate a heterozygous strain and was subsequently grown in YCB-BSA to remove the flipper cassette. The resulting strain was transformed with pSKD541 to generate a homozygous deletion and was grown in YCB-BSA to generate strain SKD14.

#### Generation of strains for testing the role of HCR-Y in GFP expression

The strategy for assessing the role of HCR-Y in the regulation of GFP reporter expression was to insert a transcriptional terminator downstream of the HCR-Y transcript initiation site in the HCRc strain and monitor loss of repression of GFP expression. However, it was first necessary to identify *C*. *albicans* DNA with transcription termination properties. Transcriptional termination sequences downstream of the *C*. *albicans ADH1* gene were mapped by inserting DNA from the region downstream of the *ADH1* stop codon into the untranslated region of GFP3 (44 bp downstream of the GFP3 transcript initiation site) in plasmid HCRd. To insert *ADH1* downstream DNA into HCRd, a SmaI site was introduced at the desired insertion site as follows. Two HCRd fragments consisting of a 371 bp 5’ fragment with XhoI/SmaI ends and a 730 bp 3’ fragment with SmaI/PstI ends were amplified using plasmid HCRd as a template and primers shown in S2 Table. The 5’ and 3’ fragments were digested with XhoI and PstI, respectively, followed by digestion with SmaI. The two fragments and the plasmid backbone digested with XhoI and PstI were purified, ligated into HCRd, transformed into DH5α *E*. *coli*, screened by PCR, and verified by sequencing. PCR-generated *ADH1* fragments with SmaI ends were cloned into the SmaI-digested plasmid. SmaI sites were then removed by overlap extension PCR using primers spanning the SmaI insertion sites but lacking SmaI DNA. The resultant plasmid constructs were introduced into strain CAI4. GFP fluorescence of strains with 100 or 152 bp of *ADH1* downstream DNA was negligible under yeast growth conditions, consistent with the presence of a termination sequence, whereas introduction of 25 or 50 bp of *ADH1* downstream DNA resulted in fluorescence equivalent to that seen in the parental strain. The 100 bp sequence downstream of *ADH1* was denoted TerN. The absence of GFP mRNA was shown by RT-PCR. A sequence denoted TerC consisting of 100 nucleotides from the 5’ untranslated region of orf19.3983 was used to create a control strain in the HCRd background, which produced GFP fluorescence equivalent to that seen in strain HCRd. This sequence was chosen because of its low probability of recruiting histones.

Strains for testing the ability of HCR-Y to inhibit GFP transcription were created by inserting TerN and TerC 36 bp downstream of the transcription initiation site of HCR-Y in the HCRc background [7]. The procedure was similar to that used for creating the terminator mapping strains described above. To introduce a SmaI site at the desired location, two HCRc fragments consisting of a 358 bp 5’ fragment with XhoI/SmaI ends and a 176 bp 3’ fragment with SmaI/HindIII ends were PCR amplified using plasmid HCRc as a template and primers shown in S2 Table. The 5’ and 3’ fragments were digested with XhoI and HindIII, respectively, followed by digestion with SmaI and ligation into the plasmid backbone. The 100 bp PCR *ADH1* terminator (TerN) or control (TerC) fragments with SmaI ends were cloned into the SmaI-digested plasmid. Plasmids were transformed into *C*. *albicans* strain CAI4 to create strains HCRcTerY1 and HCRcTerY2 (containing TerN) and HCRcTerYC3 and HCRTerYC10 (containing TerC).

Constructs were verified by DNA sequencing and strains were verified by Southern blotting.

### RNA isolation and Northern blot analysis

Isolation of total RNA and Northern blot hybridization were performed as previously described [27]. Briefly, *C*. *albicans* cells were grown in YNB medium for 48 h at 25°C followed by either 2.5 h at 30°C to produce yeast forms or 2.5 h in Medium 199 at 37°C to induce hyphal forms. Cell pellets were resuspended in 1 mL of TRIzol (ThermoFisher Scientific, Carlsbad, CA) with 0.5 mm zirconia/silica beads. Total RNA was extracted as described previously [28]. 10 μg of each sample was electrophoresed through a 1.5% agarose– 0.66 M formaldehyde gel in morpholinepropanesulfonic acid (MOPS) using a running buffer consisting of 20 mM MOPS, 10 mM sodium acetate, 2 mM EDTA, pH 7.0 and then transferred to a Hybond-N^+^ membrane (GE Healthcare Life Sciences, Pittsburgh, PA).

Northern blot probes were prepared as follows: A *HWP1* probe was cut with XhoI and XbaI from plasmid pBS+13 [23]. Both GFP and 18S rRNA probes were amplified by PCR using primer sets shown in S2 Table and pSKD77 and genomic DNA as templates. The primers for GFP were GFP-start and GFP-stop, for 18S rRNA were 18S rRNA-N and 18S rRNA-C, for H41 were Yst probe DNA XhoI F and Yst probe DNA XbaI R, and for A1 were ALS3YN-XhoI-F and ALS3YN-Xba1-R. Each DNA fragment was gel-purified and radiolabeled with [gamma-^32^P]dCTP by the random-priming method (Ready-To-Go Labeling kit; GE Healthcare Life Sciences, Pittsburgh, PA). Hybridization was performed under high-stringency conditions [11], followed by washing and autoradiography.

### Strand-specific RT-PCR and PCR

The first strand cDNAs from total RNA of *C*. *albicans* strains (2 μg) were synthesized using the Superscript^TM^ III first-strand synthesis system for RT-PCR (Cat#18080–051, Invitrogen). PCR primers for specific genes are listed in S2 Table. TopTaq DNA Polymerase (Cat#200203, Qiagen, Hilden, Germany) was used for PCR. Reactions with and without reverse transcriptase (RT) and primers to detect a product from the housekeeping gene *PMA1* served as controls.

To detect ALS3-Y, first strand cDNA was produced using primer ALS3-HT1, which was followed by PCR using primers ALS3-F and ALS3-R to detect a 175 bp product. To detect HWP1-H, first strand cDNA was produced using primer H1-R, which was followed by PCR using primers H4-F and H4-R to generate a 159 bp product.

#### qRT-PCR to detect ALS3-Y and GFP

Total *C*. *albicans* SC5314 RNA (1 μg) from yeast and hyphae was reverse-transcribed into cDNA using the iScript^TM^ cDNA synthesis kit (Cat# 170–8890, Bio-Rad, Hercules, CA) and amplified by iTaq^TM^FastSYBR Green Supermix with ROX (Cat#172–5103, Bio-Rad, Hercules, CA) using specific primer sets for each target gene and the housekeeping gene *PMA1*. Fluorescence from DNA-SYBR Green complex was monitored by the ABI PRISM 7000 System (Applied Biosystems, Foster City, CA) throughout the PCR reaction. The level of target mRNA relative to the mean of *PMA1* was calculated by the comparative Ct method. *ALS3* mRNA was detected with primers ALS3-F and ALS3-R.

To detect GFP mRNA in strain SKD233 (HCRc), total RNA from cells grown under hyphal conditions was used to prepare first strand cDNA using primer GFPstop that hybridizes to the 3' end of GFP mRNA. The first PCR, using nested primer GFP109 and the abridged anchor primer (AAP) (ThermoFisher Scientific, Waltham, MA) revealed only the hypha-specific GFP transcript under hyphal growth conditions. The second PCR using GFP109 and the abridged universal amplification primer (AUAP) to amplify products from the first PCR revealed a product that was specific to yeast growth conditions and labeled HCR-Y. For strains with terminator or terminator control sequences, GFP mRNA was detected with primers GFP70-F and GFP70-R.

### 5’RACE to identify RNA’s initiating in upstream intergenic regions of *HWP1* and *ALS3*

#### 5’ RACE to detect HCR-Y

Rapid-amplification of 5′-complementary DNA ends (5′-RACE) (ThermoFisher Scientific, Carlsbad, CA) was performed according to manufacturer’s recommendations. Briefly, DNA-free RNA from cells grown under yeast conditions was prepared by treating total RNA from strain HCRc, HCRc-TerN or HCRc-TerC with DNase I (ThermoFisher Scientific, Carlsbad, CA) for 1 h at 37°C. First strand cDNA synthesis was performed by annealing the gene-specific primer, GFP-stop, to DNA-free total RNA and reverse transcribing using SuperScript™ II reverse transcriptase. After degrading the RNA with RNase Mix, the cDNA was purified using a SNAP Column. A homopolymeric tail was then added to the 3'-end of the cDNA using TdT and dCTP. The first PCR was performed with the Abridged Anchor Primer (AAP) and GFP109 (S2 Table) using dCTP-tailed cDNA as a template. Reamplification was performed with the Abridged Universal Amplification Primer (AUAP) and GFP109 using the primary PCR products as templates. The PCR products were separated by electrophoresis through a 1.5% agarose gel and purified and cloned into pCR2.1 Topo (ThermoFisher, Carlsbad, CA) followed by transformation into TOP10 *E*. *coli*. Plasmids were purified using a QIAprep spin miniprep kit (Qiagen, Hilden, Germany) and sequenced (Geisel School of Medicine at the Molecular Biology Shared Resource, Hanover, NH).

To detect HWP1-H, RNA was prepared from strains SC5314 (*HWP1/HWP1*) and SKD14 (*hwp1∆/hwp1∆)* cultured under hyphal growth conditions. First strand cDNA was prepared using primer HHGSP1. Primers for the first and second PCR reactions were AAP and Co-H1-R, and AUAP and H4-R, respectively.

To detect RNA upstream of *ALS3*, total RNA from strain SC5314 grown under yeast or hyphal conditions was used as template to prepare first strand cDNA using primer ALS3-TR2, which is complimentary to the 3’ region of *ALS3* mRNA just downstream from the stop codon. cDNA was tailed with terminal deoxynucleotide transferase or not tailed as a control. First and second PCR reactions were performed using primers AUAP and ALS3TR1 and AUAP and ALS3R, respectively, and the products were sequenced.

#### Detection of Hwp1 on *C*. *albicans* germ tubes lacking HCR by indirect immunofluorescence

A homogeneous population of unbudded cells was prepared as previously described [29]. Briefly, cells were released into prewarmed Medium 199 at a concentration of 4 x 10^6^ cells/mL at 37°C and allowed to form germ tubes for 4 h. Cells were spun down, washed with cold PBS, and resuspended at an approximate concentration of 4 x 10^6^ cells/mL. A 200 μL aliquot of cell suspension was placed in a 1.5 mL Eppendorf tube and combined with 200 μL of primary anti-Hwp1 antibody [30] at a dilution of 1:200 in PBS. The solution was incubated at 37°C for 30 m with 100 rpm shaking. Cells were washed with 200 μL cold PBS twice prior to incubation in 200 μL secondary Ab diluted 1:50 in PBS (AlexaFluor 488 goat anti-rabbit IgG; ThermoFischer Scientific, Carlsbad, CA) at 37°C for 30 m with shaking at 100 rpm. Cells were washed twice with cold PBS and resuspended in 20 μL phenylenediamine in PBS. Epifluorescence microscopy was performed using an Olympus (Center Valley, PA) BX60 microscope and a fluorescein isothiocyanate cube with 470 to 490 nm of excitation and 515 to 550 nm of emission. Cells were photographed at 400X magnification with a ProgRes^®^ camera using MACProgRes CapturePro software (Jentopic, Jena, Germany).

#### Single cell GFP analysis to detect GFP expression in strains HCRd and HCRc

Yeast cultures of strains HCRd and HCRc were grown for 4 h in YNB and visualized with epifluorescence microscopy as described above to detect GFP. Images of 500–1000 cells were captured using a 5 s exposure and gain of 20. Single cell intensities were determined by Image J 1.50e. Frequency distributions were determined using Microsoft Excel for Mac 2011.

## Results

### HCR is important for *HWP1* expression under multiple growth conditions

To gain additional evidence for the importance of HCR in the expression of *HWP1*, we tested the effect of HCR deletion from the promoter region in the GFP reporter strain S [11] in a variety of hyphal growth conditions (Table 1). Reporter strain S is similar to strain -1902GFP except that the HCR region has been deleted. In nearly all conditions, the absence of HCR led to GFP levels that were less than 10% of those seen in strain -1902GFP. HCR was also required for *HWP1* expression on germ tubes during biofilm formation. We further confirmed the role of HCR in coupling *HWP1* expression to morphology by creating additional strains similar to HCRENO, where HCR is placed upstream of the *ENO1* promoter and regulates a GFP reporter under hyphal and yeast growth conditions as previously described [11]. GFP expression was 10 to 20 fold higher than in the vector control in these strains under germ hyphal conditions. Overall, these results confirmed that HCR is useful for determining the molecular mechanisms of repression and activation governing *HWP1* expression during the yeast/hypha transition.

**Table 1:** Role of HCR in reporter gene activation in different growth conditions.

	Fluorescence relative to strain -1902 (%)	
Growth Condition	Strain S	Strain 0GFP3 (Vector Control)
Medium 199	8.7 +/- 0.5	2.7 +/- 0.0
Medium 199 in CO_2_	5.0 +/- 0.2	2.4 +/- 0.0
YPD + Serum	9.3 +/- 0.2	2.7 +/- 0.1
Lee’s Medium, pH 6.8	7.9 +/- 1.0	3.0 +/- 0.1
Biofilm	6.5 +/- 0.9	1.0 +/- 0.8
Saliva	22.1 +/- 0.1	3.3 +/- 0.2
N-acetylglucosamine	7.8 +/- 0.2	3.3 +/- 0.3

GFP reporter strains carrying the entire *HWP1* promoter (strain -1902), the *HWP1* promoter with HCR deleted (strain S7) [11], or no promoter (strain 0GFP3) [31] were incubated under various conditions as described in Materials and Methods. The relative fluorescence of -1902 generally ranged from 40,000 to 60,000 after subtracting values for the vector control. Results are representative of a minimum of two experiments.

### Mapping regions in HCR that couple gene expression to morphology

**Fig 1:**
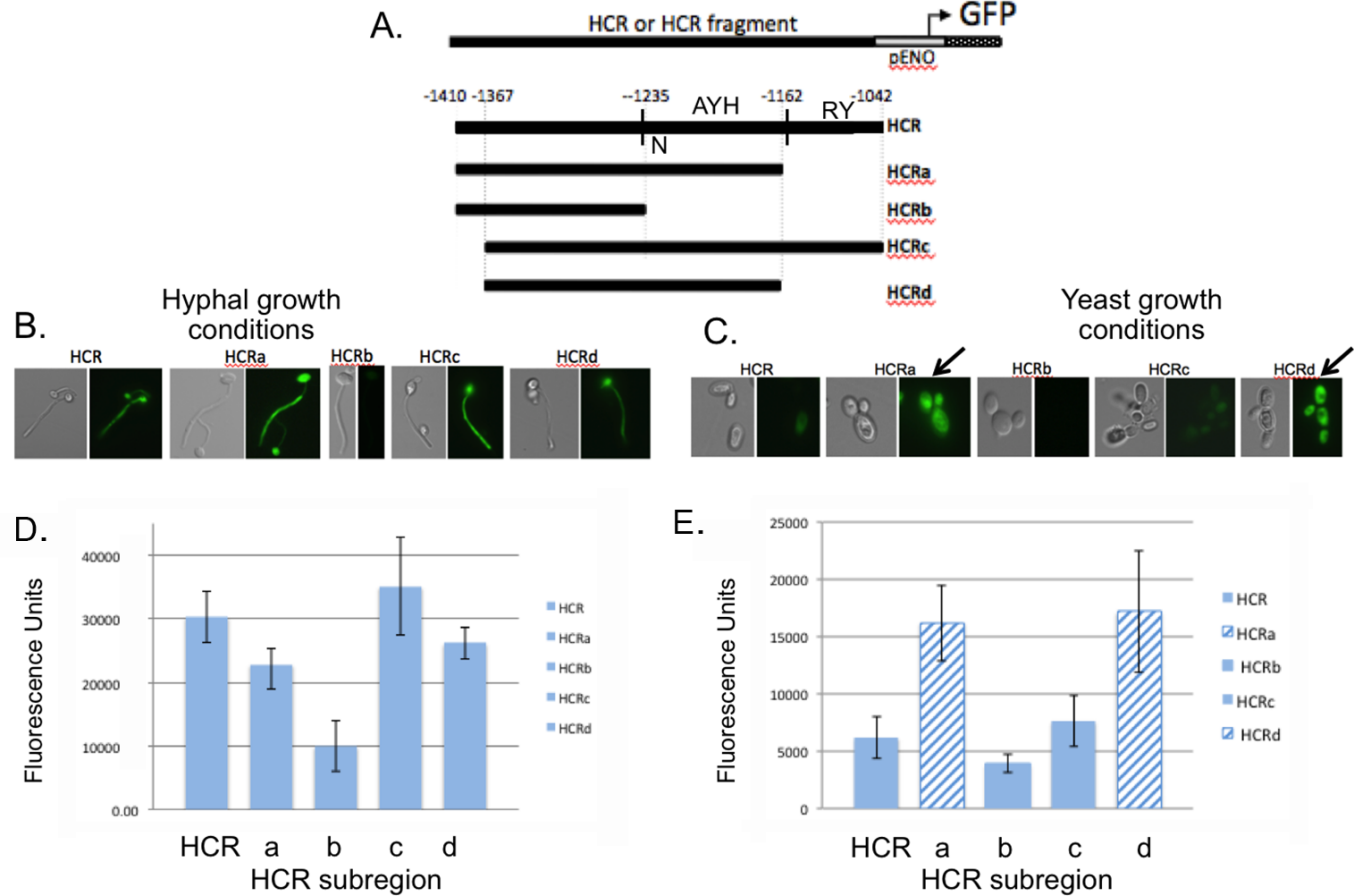
Mapping functional regions of the *HWP1* control region (HCR) by measuring GFP levels in reporter strains. (A) Diagram of constructs used to localize regions of HCR that co-regulate GFP expression with morphology. The location of the Nrg1p binding site is labeled “N”. The region between -1235 and -1162 required for activation under yeast and hyphal conditions is labeled “AYH” for activating under yeast and hyphal growth conditions. The region between -1162 and -1042 required for repression conditions is labeled “RY” for repression under yeast growth conditions. (B) and (C) Photomicrographs of strains with GFP expression driven by various HCR subregions under hypha-inducing or yeast-inducing growth conditions, respectively. (D) and (E). Fluorometry using the same strains described in (B) and (C). HCR subregions HCRa and HCRd lack the 3' terminal region encompassing -1162 to -1042 and are derepressed for GFP expression (see arrows in (C) and hatched bars in (D2)). (S1 Appendix).

To gain insight into the molecular mechanisms involved in coupling *HWP1* gene expression to morphology, we sought to identify HCR binding proteins using gel shift experiments [11]. These experiments were performed using a truncated HCR fragment termed HCRa [11] that lacks 120 bp at the 3’ end of HCR. Previous work suggested that HCRa showed the same DNA/protein binding pattern on non-denaturing gels as HCR; however, the ability of HCRa to couple GFP reporter expression to morphology had not been previously determined. To compare HCRa and other HCR fragments to the full HCR region with respect to promoter function, we created a family of *C*. *albicans* strains with HCR fragments driving the basal *ENO1* promoter upstream of a GFP reporter to compare fluorescence levels to those seen with the full HCR in strain HCRENO (Fig 1A). GFP activity was compared under hyphal growth conditions by fluorescence microscopy (Fig 1B and 1C) and fluorometry (Fig 1D and 1E). HCRa and HCRd, which consisted of the central region of HCR between -1366 and -1162, activated GFP expression in both yeast and hyphal growth conditions. Truncation of HCRa downstream of –1235 in strain HCRb led to loss of expression in both yeast and hyphal growth conditions, indicating an important role for sequences between -1235 and -1162 in gene activation under yeast and hyphal growth conditions. This region was denoted AYH (Activating and Hyphal conditions). Strain HCRc, like HCR, was repressed under yeast growth conditions. To confirm the differences in the levels of GFP fluorescence between strains HCRd and HCRc under yeast growth conditions, the fluorescence of individual cells was determined by fluorescence microscopy. All of the HCRd cells exhibited fluorscence intensity units of 60 or greater whereas less than 5% of the cells of strain HCRc fluoresced above 60 units (S1 Fig). These results are consistent with the fluorometric analysis of the entire population, indicating that HCRd activity is derepressed under yeast conditions. Because the 3’ terminal 120 base pairs of HCR are important for mediating repression during yeast growth, this region was denoted RY (Repression under yeast growth).

### The transcription factors Nrg1 and Efg1 contribute to repression and activation of gene expression through the HCR region

**Fig 2:**
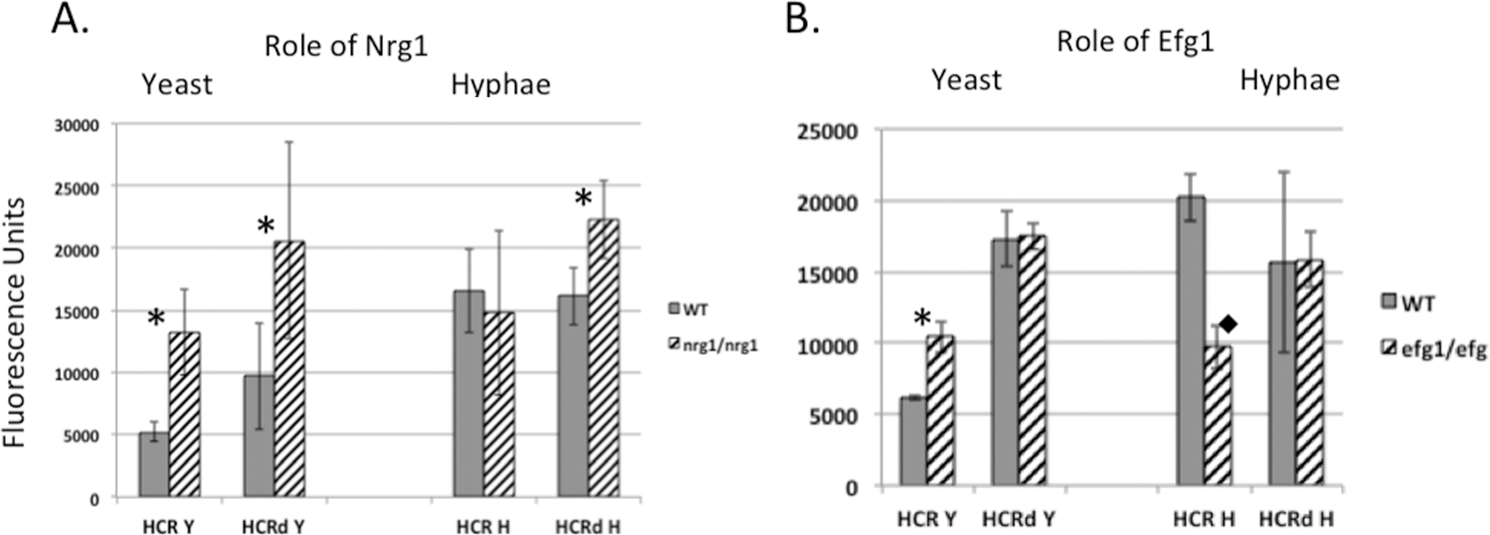
Role of Nrg1p and Efg1p in GFP reporter expression through HCR and HCRd. GFP reporter constructs under the control of HCR or HCRd were introduced into *nrg1∆/nrg1∆* to create strains SKD502 and SKD501, respectively, (A) and *efg1∆/efg1∆* mutants to create strains SKD504 and SKD503, respectively (B) as described in Materials and Methods. HCR and HCRd were also introduced into the wild type background, creating strains SKD246 and SKD245, respectively. (A) GFP expression driven by both HCR and HCRd was increased in the *nrg1∆/nrg1∆* mutant compared to the wild-type strain under yeast growth conditions. Under hyphal growth conditions, HCRd driven GFP expression was increased slightly in the *nrg1∆/nrg1∆* mutant when compared to expression in the wild-type strain. (B) Under yeast growth conditions, GFP expression driven by HCR but not HCRd was increased in the *efg1∆/efg1∆* mutant relative to wild-type. Under hyphal growth conditions, GFP expression driven by HCR but not HCRd was decreased in the *efg1∆/efg1∆* mutant relative to wild-type. Three experiments were performed in duplicate, using five independent transformants for each experiment. Statistical analysis was performed using the Student's t-test. (*indicates P<0.01. diamond indicates P<0.02). (S2 Appendix).

Given that Nrg1 has been shown to be critical for repression of *HWP1* during yeast growth conditions [13], we wondered if Nrg1 repressed expression via HCR and, if so, if repressive activity was localized to the RY region associated with HCR-mediated repression shown in Fig 1. To investigate this, we introduced the HCR and HCRd GFP reporter constructs into both the *nrg1∆/nrg1∆* mutant [20] and the wild-type strain and compared them for GFP expression. If Nrg1p’s repressive activity was localized to the region of HCR-mediated repression, then HCR but not HCRd would be derepressed in the *nrg1∆/nrg1∆* mutant compared to wild-type cells. The results showed that HCR constructs were indeed significantly derepressed in the *nrg1∆/nrg1∆* mutant strain as compared to wild-type, supporting a role for Nrg1p in repressing HCR (Fig 2A). However, HCRd was also derepressed in the *nrg1∆/nrg1∆* mutant compared to wild-type, suggesting that the HCRd region may be sufficient for Nrg1p-mediated repression and thereby revealing an additional repressor function region for (RY) between -1162 and -1042. Derepression of HCRd conditions is consistent with the presence of a predicted Nrg1p binding site in HCRd at position -1223 to -1228 (consensus sequence MVCCCT). Under hyphal growth conditions, Nrg1p appeared to repress expression slightly through HCRd as shown by the increased expression of HCRd in the *nrg1∆/nrg1∆* mutant compared to wild-type.

Since Efg1 has been shown to activate *HWP1* expression [9], we also asked if Efg1p acted through HCR. We introduced HCR and HCRd GFP reporter constructs into the *efg1∆/efg1∆* mutant [32] and compared GFP expression to the constructs in the wild-type strain background (Fig 2B). The results show that Efg1p functions through HCR in both yeast and hyphal growth conditions; however, under yeast growth conditions Efg1p exhibits repressive activity whereas under hyphal growth conditions Efg1p activates GFP expression through HCR. The wild-type and *efg1∆/efg1∆* strain backgrounds do not differ in expression in the HCRd constructs. This result indicates that Efg1p functions outside of the HCRd subregion and most likely acts through RY (-1162–-1042), although it is possible that sequences between -1410 and -1367 may be important for Efg1p activity. Taken together with observations using the *nrg1∆/nrg1∆* mutant, these results show that Nrg1p functions within DNA common to both HCRd and HCR while Efg1p functions in both yeast and hyphal growth conditions outside of HCRd.

### HCR is critical for activating native *HWP1* mRNA expression

**Fig 3:**
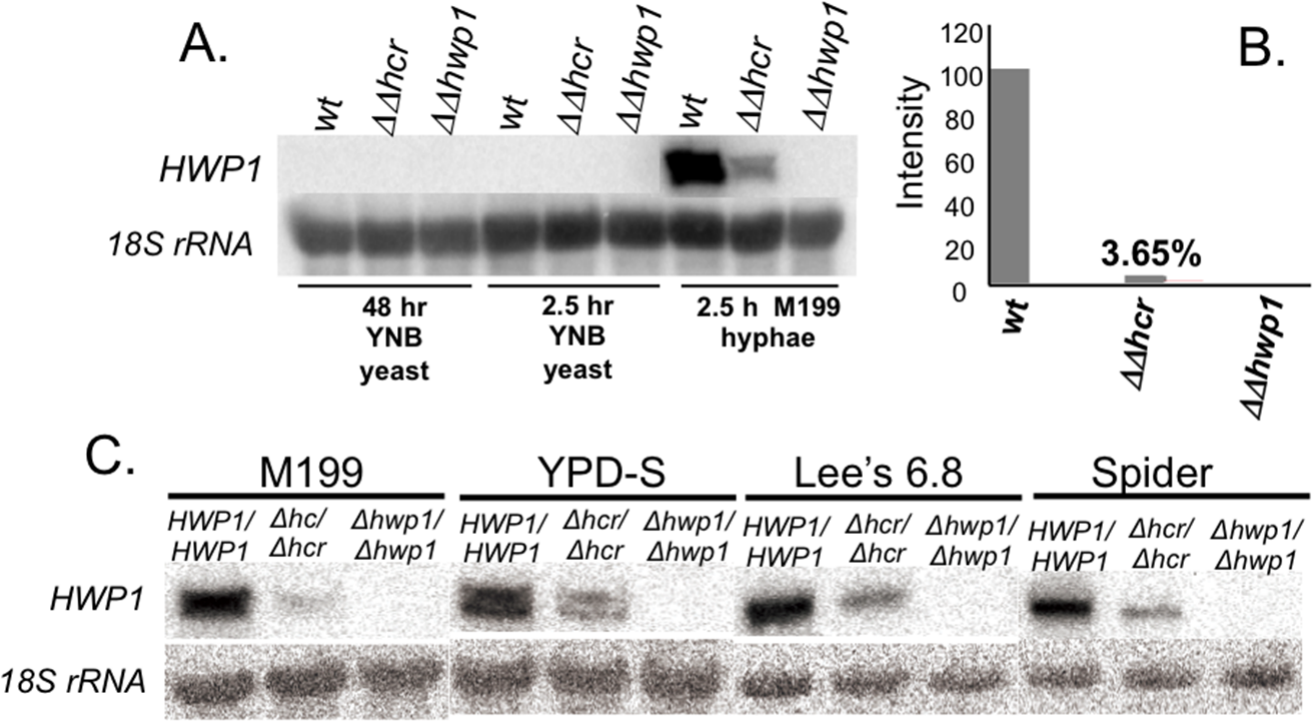
Role of HCR in *HWP1* mRNA expression. (A) Northern blot of total RNA extracted from *C*. *albicans* strains CAI4 (*HWP1/HWP1*), SKD4 (hcr∆/hcr∆), and SKD14 (*hwp1∆/hwp1∆*), under yeast and hyphal growth conditions. *HWP1* was detected with probe pBS+13 [23]. (B) Densitometry of the Northern blot shown in (A) from cells grown under hyphal growth conditions. (C) Northern blot showing reduced expression of *HWP1* in the absence of HCR using a variety of growth media known to promote *C*. *albicans* hyphal development.

To investigate how HCR affects expression at the native *HWP1* locus, we created strain SKD4 in which HCR is deleted upstream of both chromosomal copies of *HWP1*. A control strain, SKD14, contained a deletion of the entire *HWP1* locus, including upstream and downstream DNA. Northern blotting and densitometry showed that *HWP1* mRNA was reduced by over 95% in strain SKD4 compared to the wild-type strain CAI4 (Fig 3A and 3B) when grown in Medium 199 and was also markedly reduced using other growth media (Fig 3C). The need for HCR to generate wild-type levels of *HWP1* mRNA was consistent with the importance of HCR previously found using the GFP reporter strain without the HCR region (strain S) in which GFP fluorescence was reduced by over 90% under most growth conditions. These results demonstrated that HCR is important for activating full expression at the native *HWP1* promoter during hyphal growth conditions. *HWP1* is not expressed under yeast growth conditions in strain SKD4, indicating that absence of *HWP1* is HCR independent in this strain.

### Role of HCR in activating Hwp1p expression

**Fig 4:**
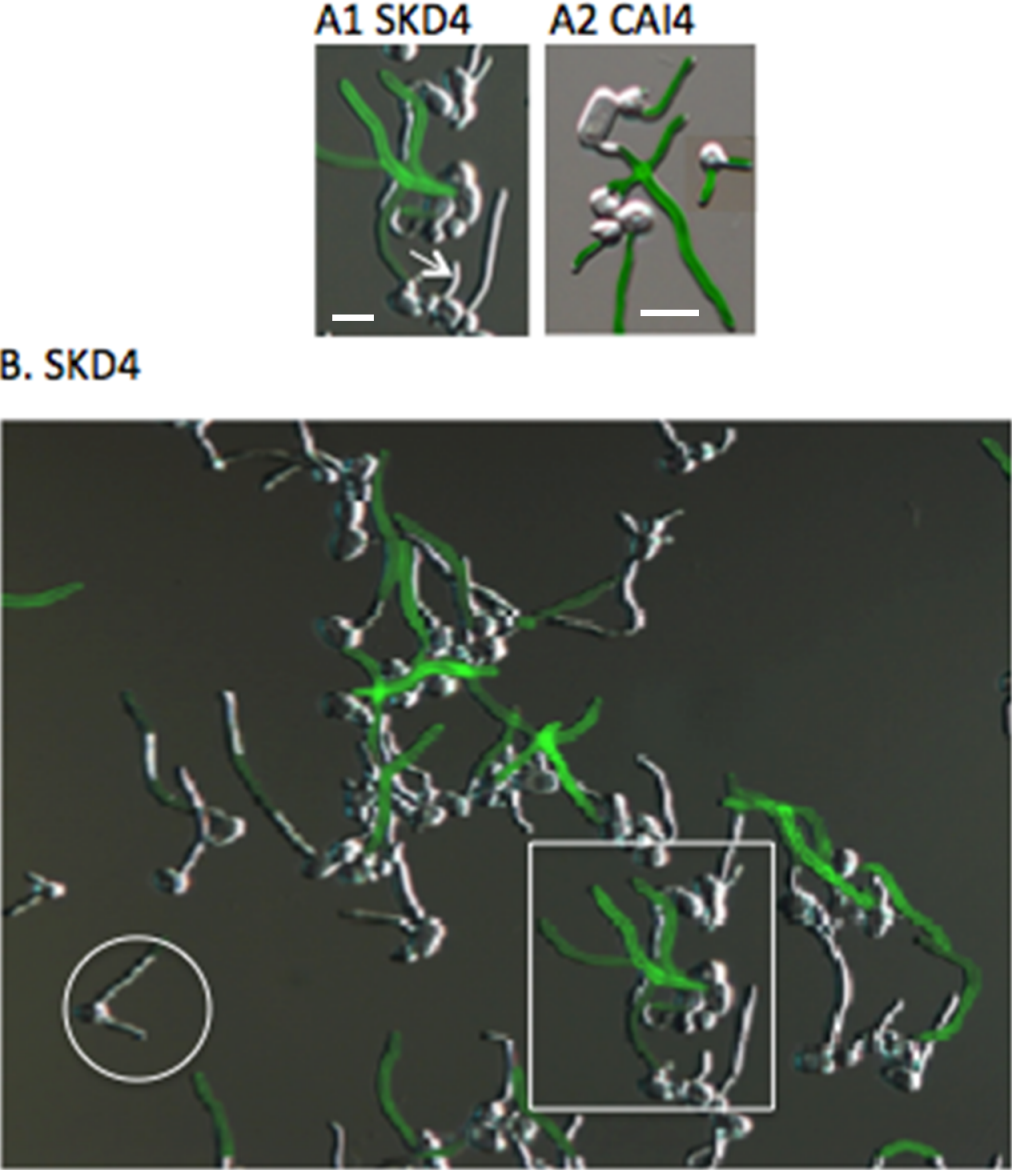
Effect of HCR deletion on Hwp1p expression. (A1) Indirect Immunofluorescence assay (IFA) of strain SKD4, which lacks the HCR upstream of both copies of *HWP1*, showing variable expression of Hwp1p on germ tube surfaces. Emerging germ tubes (white arrow) were negative for Hwp1p whereas longer germ tubes were variably positive or negative. (A2) IFA of the wild-type strain CAI4 in which germ tubes of all lengths were positive for Hwp1p. (B) Variable expression of Hwp1p on germ tubes in the cell population of strain SKD4 showing undetectable fluorescence on some germ tubes and strong fluorescence on others. The boxed area is enlarged in (A1). Scale bars are 10 microns.

To determine the effect of HCR deletion on Hwp1p expression, indirect immunofluorescence assays were performed using anti-Hwp1p antibody as previously described [33]. We expected to find very little Hwp1p expression based on the low level of *HWP1* mRNA in strain SKD4 compared to the wild-type strain. Surprisingly, Hwp1p was relatively abundant on a fraction of SKD4 germ tubes (Fig 4A1 and 4B). This result suggests the presence of stochastic mechanisms that control Hwp1p expression among germ tubes in a given cell population. The data also suggests that HCR functions to induce expression of Hwp1p uniformly on all emerging germ tubes in the population. Only 13% of emerging germ tubes of strain SKD4 expressed Hwp1p, whereas all emerging germ tubes of strain CAI4 were Hwp1p-positive as previously observed [34]. Although HCR is required for the majority of *HWP1* gene expression, these results suggest the presence of HCR-independent mechanisms of Hwp1p expression.

### Detection of a yeast condition-specific transcript in HCR

*C*. *albicans* genes with promoter regions that are much larger than the median length of 623 base pairs [35] have been shown to generate regulatory mRNAs with long 5’ UTRs [36] [37]. Although the mature *HWP1* transcript has a short 5’ UTR of 57 nucleotides, [23] we wondered if longer mRNA isoforms might be initiated from within the upstream intergenic region.

**Fig 5:**
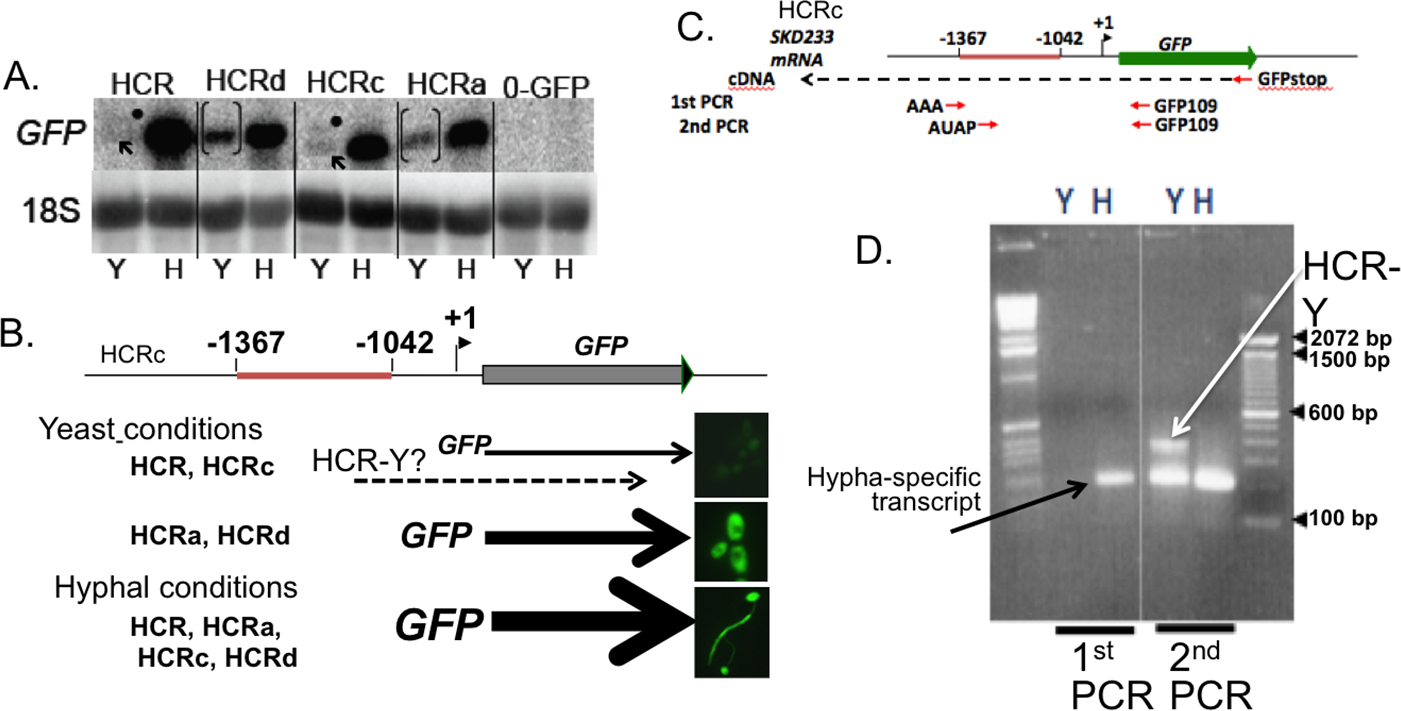
Characterization of a yeast condition-specific RNA initiating in the repressive region of HCR. (A) Northern blot showing that under yeast growth conditions, a yeast condition specific GFP mRNA (see "•") is seen along with a faint band representing the mature GFP transcript in HCR and HCRc (see "↖"). Derepression of the mature GFP transcript occurs in both HCRd and HCRa (see band inside brackets“[]”). Identical gels run and transferred to membranes on the same day were probed for HCR or 18s RNA with exposure times of 10 or 2 m, respectively. Differences in intensities of 18s band between strains could have resulted from slight differences in sample loading efficiencies, or transfer efficiencies between gels. (B) Models of the GFP transcripts present in strains HCR, HCRa, HCRb, and HCRd. The mature GFP mRNA is shown by a solid arrow with the relative abundance indicated by the thickness of the arrow. The putative yeast-specific transcript is shown as a dotted line denoted HCR-Y. Single cell images of GFP fluorescence of strains for each model are shown to the right. (C) Schematic diagram of the 5' RACE used to detect yeast specific transcripts. (D) 5' RACE used to characterize the yeast-specific transcript from strain HCRc (SKD233).

To search for transcripts associated with HCR, we examined *C*. *albicans* GFP reporter strains under the control of HCR and truncated HCR fragments by Northern blotting (Fig 5). Under hyphal growth conditions, a single mature GFP transcript was uniformly identified in strains in which GFP was controlled by HCR, HCRa, HCRc or HCRd. Under yeast growth conditions, a single mature GFP transcript was also present in strains HCRa and HCRd (bracketed in Fig 5A) that was partially derepressed under yeast conditions. However, in strains HCR and HCRc, which have been shown to repress GFP under yeast growth conditions, two faint transcripts were identified when examined under yeast growth conditions. One transcript was identical in size to the mature GFP construct while an additional faint transcript of higher molecular weight was also detected that was more distinct in HCRc than in HCR (see dots in Fig 5A) The vector control, strain 0–14, did not generate any discernable GFP transcripts. To further characterize these yeast condition specific transcripts, we initially examined strain HCRc for the presence of transcripts in the same orientation as the GFP transcript. A diagram of the GFP-related transcripts in the various HCR strains is shown in Fig 5B. The putative yeast condition specific transcript is denoted HCR-Y. To detect yeast specific transcripts, total RNA from strain HCRc was isolated and used to perform Rapid Amplication of 5’ cDNA Ends (5’ RACE system) (Fig 5C and 5D). The first RACE PCR generated a hypha-specific band representative of mature GFP mRNA, but PCR products were not detectable in yeast condition RNA. A second PCR was performed for increased sensitivity using the products of the first PCR as a template. The HCR-Y transcript was detected in the second PCR while a faint band representing the mature GFP transcript was also seen. The location of the 5’ end of HCR-Y was found to be 51 bp upstream of the 3’ end of HCR as determined by TA cloning [38] and DNA sequencing of HCR-Y. Thus, HCR-Y is initiated within the region that was found to be important for repression under yeast growth conditions as shown in Fig 1. These results are consistent with the hypothesis that repressive activity located within HCR is associated with the initiation of an unstable transcript with the same orientation as the downstream GFP reporter gene.

**Fig 6:**
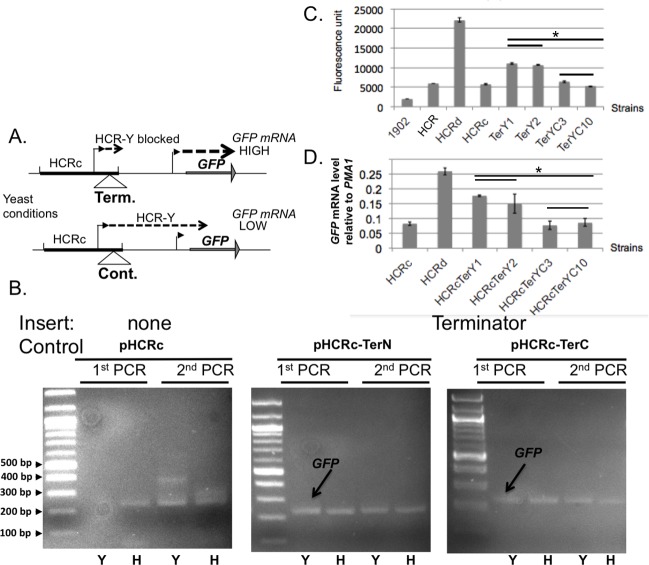
Transcriptional termination experiment to test the role of HCR-Y in interfering with GFP expression under yeast growth conditions. (A) Experimental strategy to block transcription of HCR-Y under yeast growth conditions. A transcriptional termination sequence from the *ADH1* gene was inserted downstream of the HCR-Y transcript initiation site to create strain pHCRc-TerN. Control DNA was used to create strain pHCRc-TerC. (B) 5’ RACE analysis suggesting reciprocal expression of GFP and HCR-Y under yeast growth conditions. (C) and (D) GFP expression under yeast growth conditions determined by fluorometry to detect protein (C) and qRT-PCR to detect mRNA (D). Statistical analysis was performed using the Student's t-test. (*indicates P<0.005). (S3 Appendix and S4 Appendix).

Given that the HCR-Y transcript is initiated within the repression region of HCR, we wondered if HCR-Y might play a role in suppressing *HWP1* expression under yeast growth conditions. To test this hypothesis, we attempted to block HCR-Y expression under yeast growth by placing a transcriptional termination sequence between the HCR-Y initiation site and the *ENO1* promoter. A transcriptional termination sequence downstream of the *ADH1* gene along with a control sequence was identified for this purpose as described in Materials and Methods. A diagram of the experimental strategy is shown in Fig 6A. The transcriptional termination sequence blocked expression of HCR-Y as determined by 5’ RACE and increased the amount of the mature GFP-H transcript conditions in the first PCR compared to strains lacking inserts or having the control insert (Fig 6B, black arrow). qRT-PCR and fluorometry (Fig 6C and 6D) showed that GFP mRNA and protein were significantly increased compared to HCRc, suggestive of a role for HCR-Y in transcriptional repression. However insertion of the terminator did not fully derepress GFP mRNA and protein to the level of HCRd. Strains where control DNA was inserted at the same location retained repression of GFP mRNA and protein equivalent to HCRc and HCR, consistent with the lack of termination sequences in the control DNA. Unexpectedly, HCR-Y was not detectable by 5’ RACE in the control strain (Fig 6B, right panel). The reasons for this are unknown. Perhaps HCR-Y was present but less stable as a consequence of the inserted DNA and therefore not discernible by 5’RACE. Overall, these results are consistent with the involvement of HCR-Y in suppressing activation from the HCR region by transcriptional termination. However, the inserted DNA may have interfered with the activating function within HCRd, preventing full derepression of GFP expression.

### Detection of a hypha-specific mRNA isoform at the *HWP1* locus

**Fig 7:**
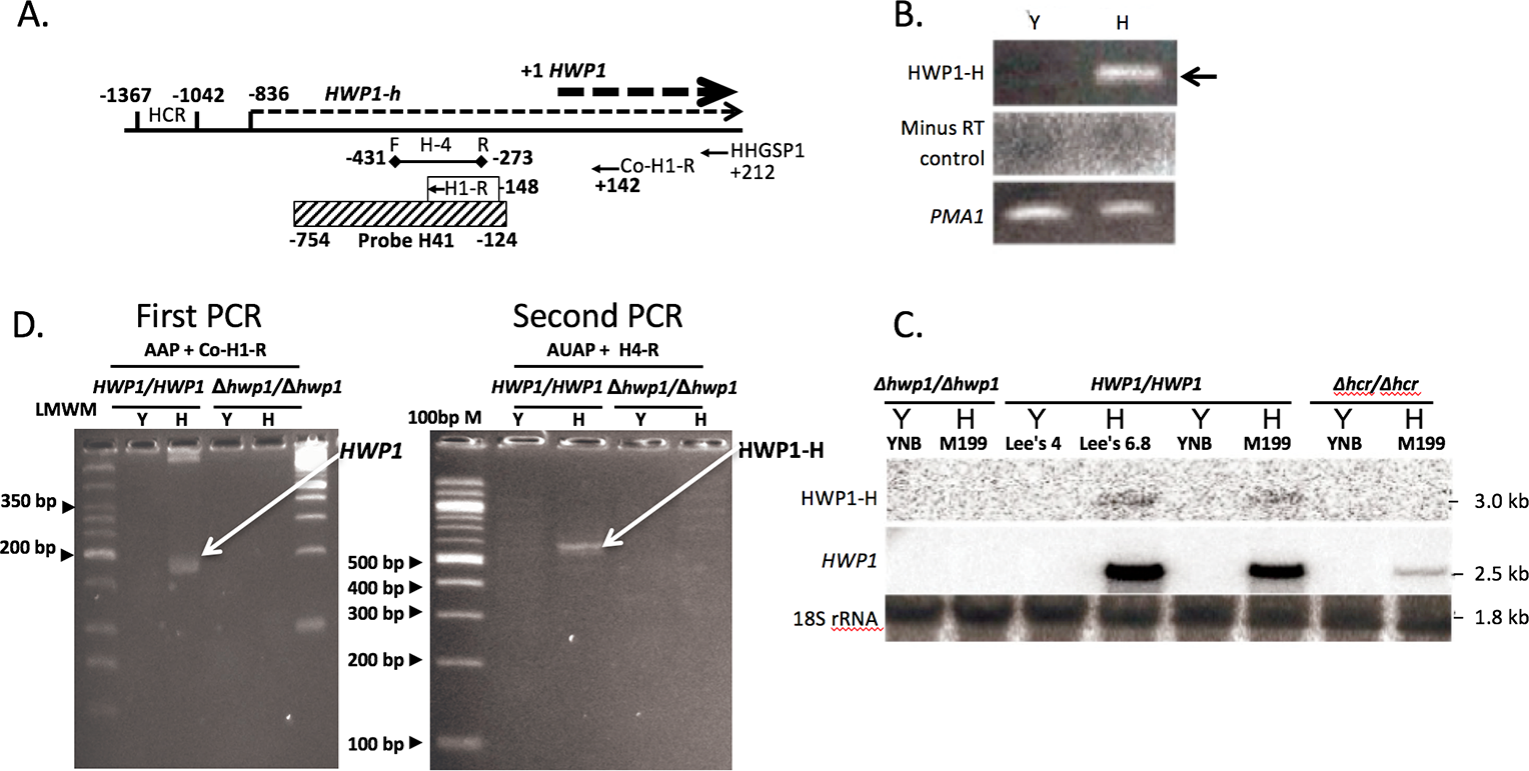
Detection of an mRNA isoform initiating upstream of the *HWP1* transcription start site under hyphal growth conditions. (A) Schematic diagram of the region upstream of *HWP1* showing the locations of the HWP1-H initiation site and the primers and probe used for its detection. The numbers are relative to the *HWP1* transcription initiation site (denoted +1), which is 57 bp upstream from the translation start site [23]. Transcripts are indicated with dotted lines. (B) Detection of HWP-H by strand-specific RT-PCR. (C) Northern blot prepared using total RNA from strains SKD14 (*hwp1∆/hwp1∆)*, CAI4 (*HWP1/HWP1*) and SKD4 (hcr∆/hcr∆) probed with H41 (hatched rectangle in (A)) to detect HWP1-H and pBS+13 [23] to detect *HWP1*. HWP1-H was detected under hyphal growth conditions in the wild-type strain but not in the hcr∆/hcr∆ or *hwp1∆/hwp1∆* strains. (D) Localization of the HWP1-H initiation site by 5’ RACE. The first PCR resulted in a product derived from mature *HWP1* mRNA (arrow). The second PCR reaction generated a PCR product derived from HWP1-H (arrow). The initiation site was found to be 836 bp upstream of the *HWP1* transcription start site by DNA sequencing. HWP1-H was not detected under yeast growth conditions or in a strain in which the entire *HWP1* locus was deleted.

While investigating the presence of HCR-Y at the *HWP1* locus, we detected a hypha condition-specific transcript in the same orientation as *HWP1*. The detection of transcripts initiating in the upstream intergenic region conditions is of interest because of their potential for activating gene expression. A diagram showing the initiation site of the transcript relative to the mature *HWP1* transcript is shown in Fig 7A. The transcript, denoted HWP1-H, was detected by strand specific PCR (Fig 7B) and Northern blotting (Fig 7C) in RNA from the wild-type strain under hyphal growth conditions induced using Lee’s medium at a pH of 6.8 as well as Medium 199. The transcript was absent in strain SKD14 in which the entire *HWP1* locus was deleted. HWP1-H was found to be initiated 836 bp upstream of the mature *HWP1* mRNA as determined by DNA sequencing of 5’ RACE products (Fig 7D). The presence of HWP1-H appeared to depend on HCR since it was absent in strain SKD4, which lacks HCR (Fig 7C). HWP1-H was also not detectable in strain S, which lacks HCR, and was detectable in strain 1902 using 5’ RACE, consistent with HCR being required for *HWP1-H* expression (not shown).

### Yeast and hyphal growth condition-specific mRNA upstream of *ALS3*

**Fig 8:**
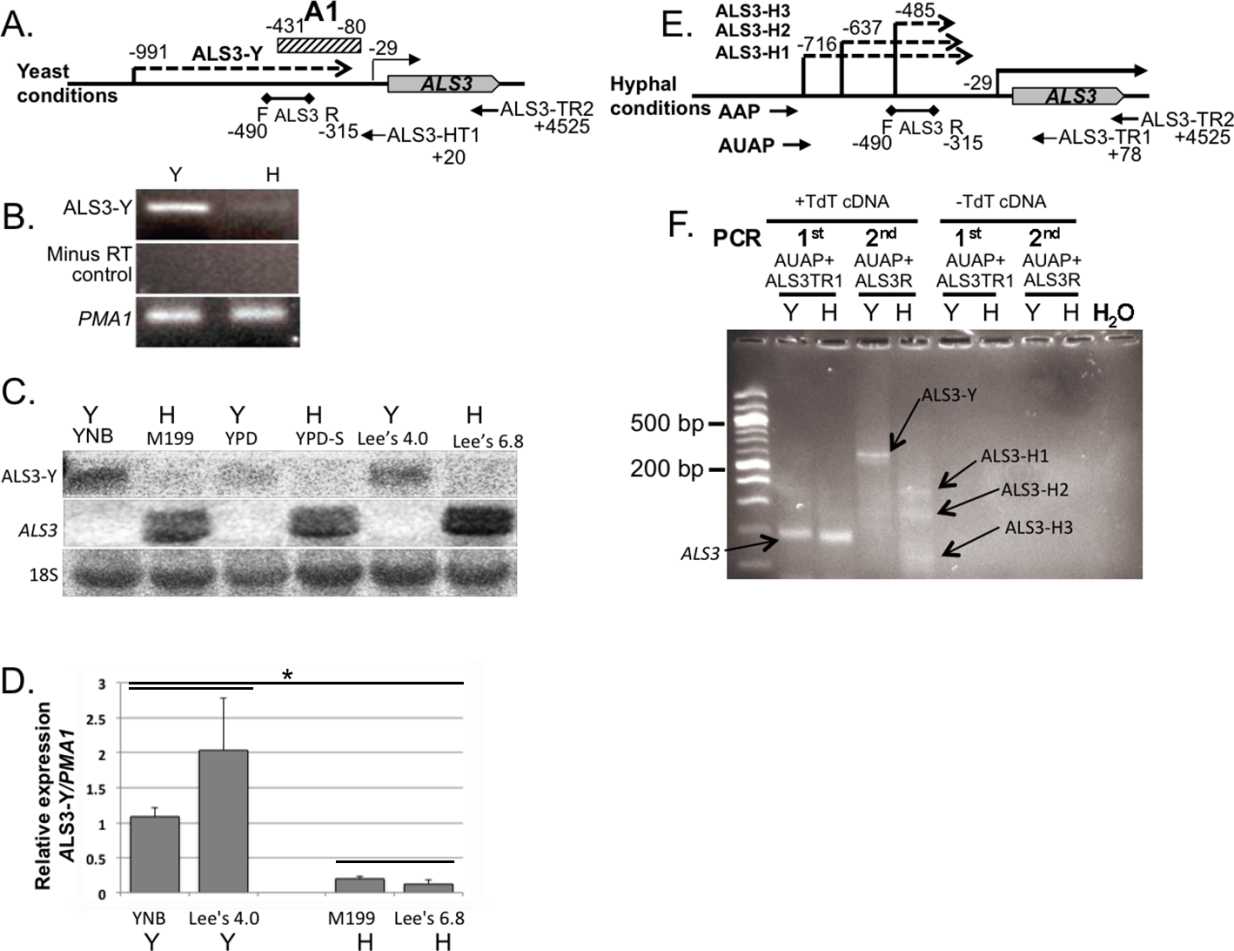
Detection of yeast and hyphal growth condition-specific RNAs initiated upstream of *ALS3* in strain SC5314. (A) Schematic diagram of the DNA upstream of *ALS3* showing the location of the ALS3-Y initiation site during yeast conditions and the primers and probe used for detection. The numbers are relative to the *ALS3* translation start site (denoted +1). (B) Detection of ALS3-Y by strand specific PCR. (C) Northern blot for detection of ALS3-Y. RNA from strain SC5314 was probed with DNA fragment A1 (hatched rectangle in (A)) spanning the region from -431 to -80 bp upstream of the *ALS3* translation start site. The membrane was exposed for three weeks. (D) qRT-PCR showing increased abundance of ALS3-Y under yeast growth conditions compared to hyphal growth conditions. Error bars represent the standard deviation from the mean. The fold change in the amount of the ALS3-Y transcript and hyphal conditions was significant as determined by the Student’s *t* test (*indicates P <0.005) S5 Appendix (E) Schematic diagram of the DNA upstream of *ALS3* showing the locations of the initiation sites of ALS3-H1, ALS3-H2 and ALS3-H3 under hyphal growth conditions and the primers used for their detection. (F) 5’ RACE to detect the initiation sites of RNAs upstream of *ALS3*. A PCR product derived from the mature *ALS3* transcript was detected in the first and second PCR reactions. DNA sequencing revealed the start site location of ALS3-Y to be -991 bp upstream of the translation start site (A). Three transcripts were detected under hyphal growth conditions in the second PCR reactions. DNA sequencing of the PCR products revealed start sites at -716, -637, and -485 for ALS3-H1, ALS3-H2 and ALS3-H3, respectively.

To investigate the possibility of RNAs initiating in upstream regions of hypha-specific genes and functioning in regulating gene expression in general, we analyzed the *ALS3* locus. *ALS3* encodes another hypha-specific protein with multiple properties, including adherence and invasion [39]. Analysis of published transcriptome data [16, 36, 40] revealed the potential presence of a stable yeast condition-specific transcript upstream of *ALS3* (Fig 8A). We confirmed the transcriptome results using strand-specific PCR and Northern blotting (Fig 8B and 8C). The relative levels of the putative yeast condition-specific RNA compared to hyphal conditions were measured by qRT-PCR (Fig 8D). The results were consistent with the presence of an mRNA isoform denoted ALS3-Y that was 5 to 15 fold increased under yeast compared to hyphal growth conditions depending on the particular media used for growth. To determine the initiation site of ALS3-Y, we performed 5’ RACE using RNA prepared from cells grown in both yeast and hyphal growth conditions. DNA sequencing of the products revealed the transcript initiation site of ALS3-Y to be -991 relative to the translation start site of *ALS3*. In addition, the initiation sites of three transcripts specific to hyphal growth conditions were detected at –717, -637 and -435 respectively. Taken together, these results suggest that intergenic morphology specific RNAs may not be specific to *HWP1* but rather represent a general paradigm for the regulation of growth phase-specific gene expression in *C*. *albicans*.

## Discussion

### Morphology-specific gene expression of *HWP1* is controlled by separable regions of activation and repression in HCR that are acted upon by Nrg1p, Efg1p, and unknown factors

Since its discovery two decades ago, *HWP1* [23] has become one of the most highly studied genes in *C*. *albicans* because of its abundant expression [9, 23, 41, 42], importance in virulence [2, 43–46], association with hyphal morphology, and utility in helping to decipher how fungi couple gene expression to morphology [12, 13, 33, 47]. Our functional mapping experiments of the 368 bp HCR promoter region has considerably advanced our understanding of the processes regulating morphogenic gene expression. The results elucidate how hypha-specific gene expression is activated by a release from transcriptional repression as had been suggested from previous research [8]. A central 73 bp region of HCR activates gene expression in both yeast and hyphal growth conditions as shown by comparing reporter GFP levels generated by strains HCRd and HCRb. Repression of this activation under yeast growth conditions is accomplished via two mechanisms which may or may not be interdependent. First, Nrg1p is likely to be responsible for partial repression as shown by comparing the expression of HCRd constructs in the *nrg1∆/nrg1∆* mutant to wild-type backgrounds under yeast growth conditions. This is consistent with the presence of a predicted Nrg1p binding site located within this activating region. However, full repression during yeast growth conditions requires the presence of the region denoted RY at the 3’ end of HCR as shown by comparing GFP levels of HCRc to HCRd via fluorometry and fluorescence microscopy to study populations of cells and individual cells, respectively. The repressive activity of RY under yeast growth conditions may be mediated by Efg1p as shown by comparing the activity of HCR and HCRd in the *efg1∆/efg1∆* mutant and wild-type backgrounds; however, sequences upstream of HCRd in HCR may also be acted upon by Efg1p. Thus, both Nrg1p and Efg1p are important for repressing *HWP1* expression via HCR during yeast growth conditions. Under hyphal growth conditions, Efg1p is important for activating gene expression, but its activity is not associated with the central 73 bp region of HCR as described above, but rather to regions outside of HCRd in similar fashion to the regions mediating repression by HCR under yeast conditions. Given that Efg1's functions co-localize with repressing activity in HCR, Efg1p may function to interfere with the mechanisms causing repression that are localized to the 3’ end of HCR upon hyphal induction. This interference would result in the activation of GFP expression under hyphal growth conditions. The finding that Efg1p mediates both repression via HCR under yeast conditions and activation under hyphal conditions implicates Efg1p activity as playing a central role in coupling gene expression to morphogenesis through HCR DNA.

The repressive activity of Nrg1p on *HWP1* promoter activity probably involves direct binding of Nrg1p to its predicted binding site and recruitment of Tup1p-repressive machinery [8]. However, given that Efg1p is a master regulator that affects many other transcription factors, its interaction with HCR DNA is less certain. It is likely that the observed repressive effects of Efg1p is indirect given that HCR does not contain the Efg1p recognition sequence (EGR-motif). This motif confers DNA binding under yeast growth conditions, and Efg1p has been found to be absent from the *HWP1* gene locus conditions[48]. However, a weak peak of Efg1p binding upstream of *HWP1* has been observed using genome wide ChIP-on-chip 30 m after placing cells under hyphal growth conditions [48], suggesting that the activating role of Efg1p under hyphal growth conditions may involve direct interactions between Efg1p and HCR. Alternatively, Efg1p may activate *HWP1* expression indirectly through one or more of Efg1p’s interacting target genes.

### Potential role for mRNA isoforms in morphology specific gene expression

In the process of elucidating the function of *HWP1* gene expression through HCR, we discovered a potential general role for mRNA isoforms in the expression of hypha-specific genes. The mature *HWP1* and *ALS3* hypha-specific transcripts have short 5’ UTR’s [23, 49]. In our efforts to elucidate the mechanism of repression of the 3’ region of HCR, we identified HCR-Y as an mRNA isoform with the same orientation as the *HWP1* transcript that was specifically detected under yeast but not hyphal growth conditions. The initiation site of HCR-Y was localized within the 120 bp region denoted RY responsible for repression during yeast growth conditions, suggesting that HCR-Y may play a role in the repression of *HWP1*. Having identified HCR-Y in strain HCRc, we sought to verify its presence at the native Hwp1 locus; however, we were unsuccessful. The reasons for this are unknown but are likely related to the unstable nature of the transcript. Technical problems with primer design due to sequence similarities with other regions of the *C*. *albicans* genome may also have contributed to our inability to detect HCR-Y at the native Hwp1 locus by 5’ RACE.

A potential role for mRNA isoforms in repressing hypha-specific gene expression during yeast growth conditions was further supported by the detection of a stable yeast-specific transcript, ALS3-Y, at the *ALS3* locus. ALS3-Y was shown to be over five-fold more abundant compared to hyphal growth conditions by qRT-PCR. As for HCR-Y, ALS3-Y initiated several hundred base pairs upstream of the mature gene transcript with its short 5’ UTR found under hyphal growth conditions. The long 5’ UTR in ALS3-Y that we identified by 5’ RACE conditions is consistent with results found from transcriptome profiles of the *C*. *albicans* genome [16, 36, 40].

mRNA isoforms with extended 5’UTR’s specific to hyphal growth conditions were also found upstream of the transcript initiation sites of mature *HWP1* and *ALS3* mRNA. In the case of HWP1-H, which initiates downstream of HCR, expression was found to be dependent on the presence of HCR. This suggests that release of repression through HCR is required for activation of HWP1-H. The possibility that HWP1-H contributes to the activation of *HWP1* by interfering with Tup1p-mediated repression (*HWP1* is repressed by Tup1p [50]) is suggested by work using *S*. *pombe* in which the *FBP1* gene is activated by a cascade of intergenic RNAs that disrupt Tup1p-mediated repression [51]. Alternatively, release from repression may have an overarching effect on chromatin that opens up the DNA under hyphal growth conditions that are obscured under yeast growth conditions.

More research is required to determine if HCR-Y and ALS3-Y function in repressing *HWP1* and *ALS3* gene expression, respectively, and if so, by what means repression takes place. Recent elegant work on transcription factors *UME1* and *WOR1* have elucidated a role for their extended 5’ UTR’s in inhibiting translation [52, 53]; however, many mechanisms are possible [37]. The absence of mature *ALS3* and *HWP1* transcripts under yeast growth conditions and the instability of HCR-Y led us to favor mechanisms that did not rely on a physical activity of the 5' UTR. Prior knowledge gained using *S*. *cerevisiae* demonstrating the repressive effects of intergenic RNAs upstream of the *SER3* [54] and *FLO11* [55, 56] genes suggested that transcriptional interference mediated by HCR-Y might also inhibit expression of *HWP1* mRNA. Blocking the expression of HCR-Y using a transcriptional terminator from the *C*. *albicans ADH1* gene led to increased expression of reporter GFP mRNA and protein, consistent with a suppressive mechanism involving transcriptional interference. However, derepression by blocking HCR-Y was not as high as derepression in the absence of the HCR-Y 3’ region (RY) using HCRd, suggesting that HCR-Y might not be responsible for the complete repression of GFP under yeast growth conditions. However, a more likely explanation for the results is that the process of blocking HCR-Y may also have interfered with the activation of gene expression as mediated by the AYH region. Further research is needed to determine if mRNA isoforms that initiate in upstream intergenic regions are important for regulating morphology-specific gene expression in *C*. *albicans*.

The presence of 5’ UTRs of different lengths and hyphal growth conditions may be indicative of mechanisms to differentiate the two morphologies as suggested by previous observations made using white- and opaque- phase cells [15, 16]. Similarly, the association of HCR-Y and ALS3-Y with yeast growth conditions may reflect the presence of an underlying mechanism to repress expression of hypha-specific genes in *C*. *albicans*. In any case, the results strongly suggest that the lengthy intergenic regions upstream of hypha-specific genes are likely important for regulating morphology-specific expression via the transcripts that are initiated within them.

### Requirement of HCR for conferring uniform expression of *HWP1* in populations of cells

An unexpected role for HCR was uncovered in experiments to determine the role of HCR in the expression of *HWP1* at its genomic locus. Deletion of HCR on both chromosomal homologues upstream of *HWP1* resulted in a dramatic reduction in *HWP1* mRNA as expected; however, visualization of Hwp1p on germ tube surfaces using an Hwp1-specific antibody revealed variable expression of Hwp1p within a single culture. This observation indicates that factors that mediate morphological transitions between yeast and hyphae act via HCR to ensure uniform activation of Hwp1p on all emerging germ tubes in a population. The variable expression of Hwp1p under hyphal growth conditions in the absence of HCR suggests the presence of stochastic mechanisms of expression of Hwp1p on germ tubes reminiscent of those described for *FLO11* in *S*. *cerevisiae* [57]. In *S*. *cerevisiae*, expression of *FLO11* is associated with pseudohyphal cells in mixed morphological populations and is regulated by promoting and interfering intergenic RNAs [55, 56]. The *S*. *cerevisiae* transcription factors that regulate the intergenic RNAs controlling expression have homologues in *C*. *albicans* [58, 59], prompting the hypothesis that *HWP1* expression in the absence of HCR may be regulated by mechanisms similar to those that regulate *FLO11* in *S*. *cerevisiae*. Variable *HWP1* expression independent of HCR helps explain why the HCR GFP reporter strain does not fully activate gene expression to the level of the entire *HWP1* upstream region [11].

In conclusion, the presence of HCR-dependent and independent expression of the *HWP1* gene strongly suggests that multiple mechanisms exist for inducing morphology specific *HWP1* expression in *C*. *albicans*. A key question for future studies is to determine the contribution of each mechanism to growth of *C*. *albicans* in the host. GFP expression in strain S, which lacks HCR, in saliva was at least two fold higher than in other media, suggesting that HCR-independent *HWP1* expression, as well as HCR-dependent expression, is important for growth in the host. The elucidation of HCR-independent *HWP1* gene expression adds additional complexity to the mechanisms leading to hypha-specific gene expression and raises questions about the impact of environmental conditions and signal transduction pathways on gene expression at the *HWP1* locus. Detailed analyses of gene expression in *HWP1* and *ALS3* are important for future studies to identify common mechanisms leading to morphology-specific gene expression. Future studies on the regulation of *HWP1* and *ALS3* gene expression are also important for understanding how *C*. *albicans* produces sufficient adhesins for the rapid attachment to and invasion of the oral epithelium, an important niche for adhesin-related virulence properties [45, 46]. Detailed analyses of *HWP1* and *ALS3* will be useful for generating a model for regulation of other morphology specific genes and may lead to the elucidation of common mechanisms of regulation that will provide new insights into inhibiting virulence properties of *C*. *albicans*.

## Supporting information

S1 Fig Single cell fluorescence of strains SKD233 (HCRc) and SKD232 (HCRd). The fluorescence intensities of HCRc strain cells ranged from 30 to 60 with over 70% of cells falling in the 30–40 group. In contrast, cells from strain HCRd ranged from 60 to 160 indicating that GFP expression is derepressed in HCRd compared to HCRc cells. The region -1162 to -1042 is implicated in repression.
(TIFF)

S1 Table Strains and plasmids used in this study. (DOCX)

S2 Table Oligonucleotides and probes. (DOCX)

S1 Appendix (XLSX)

S2 Appendix (XLSX)

S3 Appendix (XLSX)

S4 Appendix (XLSX)

S5 Appendix (XLSX)
