# Supplementary figures and images for: Release of transcriptional repression through the HCR promoter region confers uniform expression of HWP1 on surfaces of Candida albicans germ tubes
Source: PLoS One. 2018 Feb 13;13(2):e0192260. doi: 10.1371/journal.pone.0192260 (PMC5810986; doi:10.1371/journal.pone.0192260)

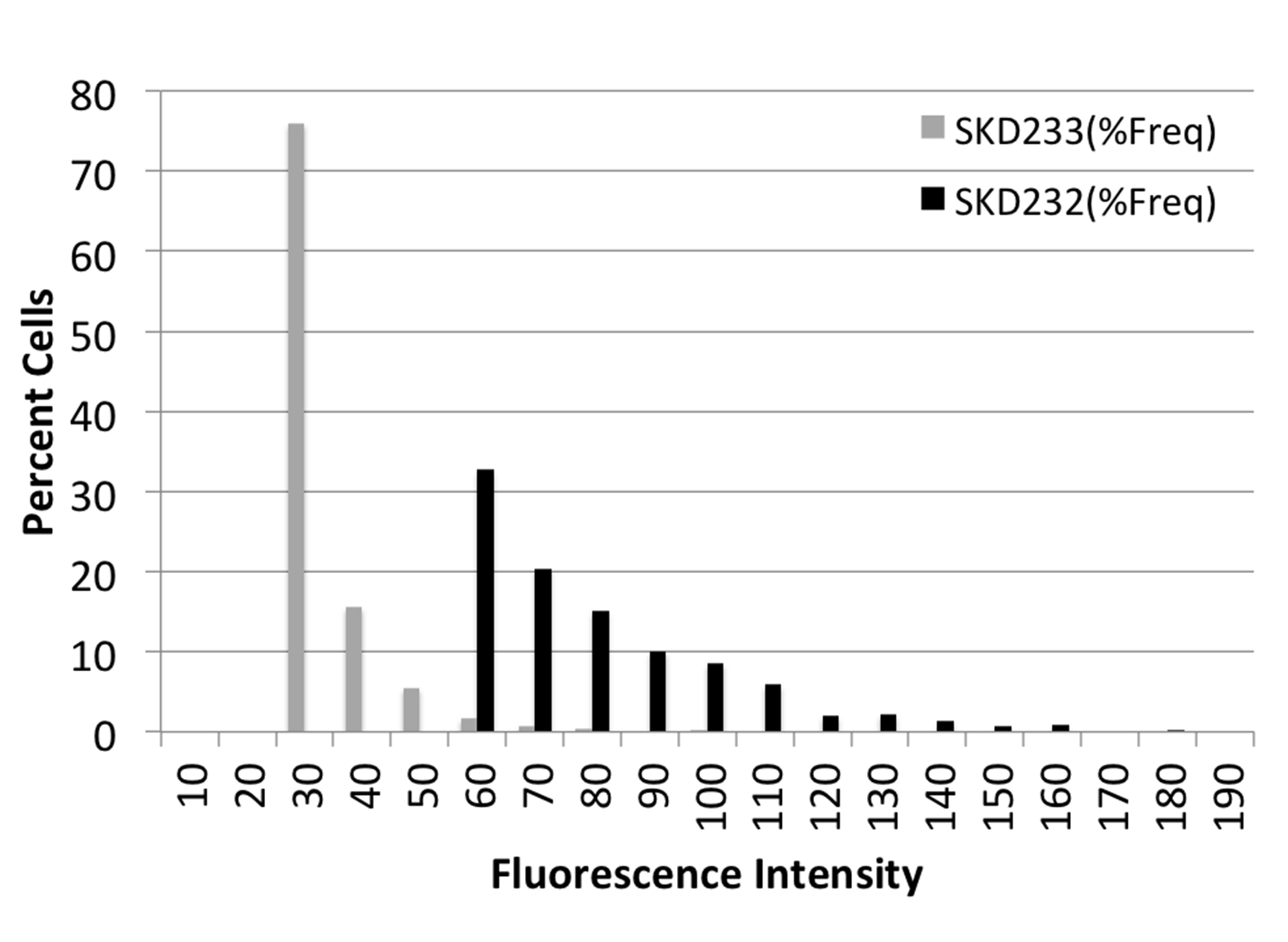

Supplement: S1 Fig — The fluorescence intensities of HCRc strain cells ranged from 30 to 60 with over 70% of cells falling in the 30–40 group. In contrast, cells from strain HCRd ranged from 60 to 160 indicating that GFP expression is derepressed in HCRd compared to HCRc cells. The region -1162 to -1042 is implicated in repression. (TIFF) [file pone.0192260.s001.tiff]
