# Supplementary material for: Release of transcriptional repression through the HCR promoter region confers uniform expression of HWP1 on surfaces of Candida albicans germ tubes
Source: PLoS One. 2018 Feb 13;13(2):e0192260. doi: 10.1371/journal.pone.0192260 (PMC5810986; doi:10.1371/journal.pone.0192260)
Supplement: S1 Table — (DOCX) [file pone.0192260.s002.docx]

**S1 Table**. Strains and Plasmids Used In This Study

| **Strain** | Genotype or description | Reference or source |
| --- | --- | --- |
| SC5314 | Wildtype | [1] |
| CAI4 | *Δura3::imm434/ Δura3::imm434* | [2] |
| 1902 | CAI4 transformed with plasmid p1902 | [3] |
| S7 | CAI4 transformed with plasmid pSGFP3 | [3] |
| 0HWP3 | CAI4 transformed with the promoterless p0GFP3 plasmid | [31] |
| HLC52 | *ura3::1 imm434/ura3::1 imm434 efg1::hisG/efg1::hisG-URA3-hisG* | [4] |
| MMC3 | *ura3::1 imm434/ura3::1imm434, nrg1::hisG-URA3-hisG/nrg1::hisG* | [5] |
| SKD231 (HCR) | CAI4 transformed with plasmid pSKD77 | This study |
| HCRa | CAI4 transformed with plasmid pHCRa | This study |
| HCRb | CAI4 transformed with plasmid pHCRb | This study |
| SKD233 (HCRc) | CAI4 transformed with plasmid pSKD78 | This study |
| SKD232 (HCRd) | CAI4 transformed with plasmid pSKD76 | This study |
| SKD4 | *Δhcr/Δhcr* (CAI4 background) | This study |
| SKD14 | *hwp1/hwp1* (CAI4 background). The CDS and surrounding intergenic DNA were deleted. | This study |
| SKD246 | CAI4 transformed with plasmid pSKD600 (HCR) | This study |
| SKD245 | CAI4 transformed with plasmid SKD599 (HCRd) | This study |
| SKD502 | *nrg1/nrg1* transformed with plasmid pSKD600 (HCR) | This study |
| SKD501 | *nrg1/nrg1* transformed with plasmid pSKD599 (HCRd) | This study |
| SKD504 | *efg1/efg1* transformed with plasmid pSKD600 (HCR) | This study |
| SKD503 | *efg1/efg1* transformed with plasmid pSKD599 (HCRd) | This study |
| HCRcTerY1 and HCRcTerY2 | CAI4 transformed with pSKD78 with *ADH1* terminator DNA inserted 36 bp downstream of the HCR-Y transcript initiation site | This study |
| HCRcTerYC3 and HCRcTerYC10 | CAI4 transformed with pSKD78 with *ADH1* terminator control DNA inserted 36 bp downstream of the HCR-Y initiation site | This study |
| **Plasmid** |  |  |
| pBS+13 | pBluescript containing 609 bp *HWP1* cut with XhoI and XbaI for Northern *HWP1* probe | [6] |
| p0GFP3 | pHWP1GFP3 digested with *Xho*I and *Hind*III to remove the *HWP1* promoter region | [31] |
| p1902 | pBluescript containing 1,902 bp upstream of *HWP1* driving GFP expression | [3] |
| pSKD77 | Partial 5’UTR of *HWP1* corresponding to -1410 to -1042 (HCR) in pENO1GFP3 | [7] |
| pSKD76 | Partial 5’UTR of *HWP1* corresponding to -1367 to -1162 (HCRd) in pENO1GFP3 | This study |
| pSKD78 | Partial 5’UTR of *HWP1* corresponding to -1367 to -1042 (HCRc) in pENO1GFP3 | This study |
| pHCRa | Partial 5’UTR of *HWP1* site -1410 to -1162 in pENO1GFP3 | This study |
| pHCRb | Partial 5’UTR of *HWP1* corresponding to -1410 to -1235 in pENO1GFP3 | This study |
| pSKD599 | Partial 5’UTR of *HWP1* corresponding to -1367 to -1136 (HCRd) in pNAT1 | This study |
| pSKD600 | Partial 5’UTR of *HWP1* corresponding to -1410 to -1042 (HCR) in pNAT1 | This study |
| pSKD593 | HCR 5’ (-1641 to -1410) and 3’ (-1042 to -913) flanking regions inserted upstream and downstream, respectively, of the flipper cassette in pSFU | This study |
| pSKD592 | HCR 5’ (-1641 to -1410) and 3’ (-1042 to -913) flanking regions inserted upstream and downstream, respectively, of the flipper cassette in pJK863 | This study |
| pSKD541 | Gene deletion of *HWP1* orf in pJK863 (primers 5’Hko SacII; 3’Hko, ApaI; 3’Hko, KpnI) | This study |

1. Gillum AM, Tsay EY, Kirsch DR. Isolation of the *Candida albicans* gene for orotidine-5'-phosphate decarboxylase by complementation of *S. cerevisiae* *ura3* and *E. coli* pyrF mutations. Mol Gen Genet. 1984;198(2):179-82.

2. Fonzi WA, Irwin MY. Isogenic strain construction and gene mapping in *Candida albicans*. Genetics. 1993;134(3):717-28.

3. Kim S, Wolyniak MJ, Staab JF, Sundstrom P. A 368-base-pair cis-acting *HWP1* promoter region, HCR, of *Candida albicans* confers hypha-specific gene regulation and binds architectural transcription factors Nhp6 and Gcf1p. Eukaryot Cell. 2007;6(4):693-709.

4. Lo HJ, Kohler JR, DiDomenico B, Loebenberg D, Cacciapuoti A, Fink GR. Nonfilamentous *C. albicans* mutants are avirulent. Cell. 1997;90(5):939-49.

5. Murad AM, Leng P, Straffon M, Wishart J, Macaskill S, MacCallum D, et al. *NRG1* represses yeast-hypha morphogenesis and hypha-specific gene expression in *Candida albicans*. Embo J. 2001;20(17):4742-52.

6. Staab JF, Ferrer CA, Sundstrom P. Developmental expression of a tandemly repeated, proline-and glutamine-rich amino acid motif on hyphal surfaces on *Candida albicans*. J Biol Chem. 1996;271(11):6298-305.

7. Staab JF, Bahn YS, Sundstrom P. Integrative, multifunctional plasmids for hypha-specific or constitutive expression of green fluorescent protein in *Candida albicans*. Microbiology. 2003;149(Pt 10):2977-86.
