# Supplementary material for: Release of transcriptional repression through the HCR promoter region confers uniform expression of HWP1 on surfaces of Candida albicans germ tubes
Source: PLoS One. 2018 Feb 13;13(2):e0192260. doi: 10.1371/journal.pone.0192260 (PMC5810986; doi:10.1371/journal.pone.0192260)
Supplement: S2 Table — (DOCX) [file pone.0192260.s003.docx]

**S2 Table. Oligonucleotides and Probes**

| **Name** | | **Purpose** | **Sequence (5’ – 3’)** | **Site(s) added** | |
| --- | --- | --- | --- | --- | --- |
| **5’ RACE analyses** | | | | | |
| GFP stop | First strand cDNA | | AAAGCGGCCGCTTATTTGTACAATTC | | NotI |
| GFP109 | 5’ RACE analysis | | GCATCACCTTCACCTTCACCGGAGAC | |  |
| AAP | 5’ RACE analysis | | GGCCACGCGTCGACTAGTACGGGIIGGGIIGGGIIG | |  |
| AUAP | 5’ RACE analysis | | GGCCACGCGTCGACTAGTAC | |  |
| UAP | 3’ RACE analysis | | GGCCACGCGTCGACTAGTAC | |  |
| HHGSP1 | First strand cDNA | | GGGTAATCATCACATGG | |  |
| Co-H1-R | 5’ RACE analysis | | GACCGTCTACCTGTGGGACAG | |  |
| ALS3-TR2 | First strand cDNA | | AAACAAAAAACAAACAAATAACAAAAATCTAAAAAGGCGACTATG | |  |
| ALS3-TR1 | 5’ RACE analysis | | ATAATTATACGTAGCAGCATTAGACCAAGTCAATGAATTAAAA | |  |
| Nested ALS3 | 3’ RACE analysis | | CCTGAAGCAGCCTTTAGTGG | |  |
| **qRT-PCR and RT-PCR** | | | | | |
| H1-R | First strand cDNA | | GGCGGATACAGGTGATACAAAGAG | |  |
| H4-F | RT-PCR | | GGAATTCGGAAATTCTGACG | |  |
| H4-R |  |  | GGTTGTGAGCCATTAGGGTTA | |  |
| ALS3-HT1 | First strand cDNA | | AATGTATATTGTTGTAGCAT | |  |
| ALS3-F | qRT-PCR and RT-PCR | | AACAACATCTTCCGCTTAGGTCGC | |  |
| ALS3-R |  |  | ATGGGGGACTCAGGGTTTGTAA | |  |
| QRTALS3 F [[61](#_ENREF_61)] | qRT-PCR and RT-PCR | | AATGGTCCTTATGAATCACCATCTACT | |  |
| QRTALS3 R [[61](#_ENREF_61)] |  |  | GAGTTTTCATCCATACTTGATTTCACA | |  |
| Coenye HWP1-F [[62](#_ENREF_62)] | qRT-PCR and RT-PCR | | GCTCAACTTATTGCTATCGCTTATTAC | |  |
| Coenye HWP1-R [[62](#_ENREF_62)] |  |  | GACCGTCTACCTGTGGGACAG | |  |
| PMA1 F | qRT-PCR and RT-PCR | | TTGAAGATGACCACCCAATCC | |  |
| PMA1 R |  |  | GAAACCTCTGGAAGCAAATTCG | |  |
| GFP70-F | qRT-PCR | | TGTTGTCCCAATTTTGGTTG | |  |
| GFP70-R | qRT-PCR | | 5ACCTTCACCGGAGACAGAAA | |  |
| **Mapping *ADH1* termination sequences** | | | | | |
| HCRd-1 | Inserting Sma1 | | CCAATCTCGAGGTGGAAATAAAG | | Xho1 |
| HCRd-2 | Inserting Sma1 | | GATCGCCCGGGAAGCTTTGTTGTAATATTCCT | | Sma1 |
| HCRd-3 | Inserting Sma1 | | GATCGCCCGGGTATTAAAATGTCTAAAGGTGA | | Sma1 |
| HCRd-4 | Inserting Sma1 | | GATCGCTGCAGTTATTTGTACAATTCATCCAT | | Pst1 |
| **Testing regulatory role for HCR-Y** |  | |  | |  |
| HCRc-1 | Inserting Sma1 | | CCAATCTCGAG GTGGAAATAAAG | | Xho1 |
| HCRc-2 | Inserting Sma1 | | GATCGCCCGGGTTACCTTTTCATTTCCTTTTT | | Sma1 |
| HCRc-3 | Inserting Sma1 | | GATCGCCCGGGTAAGAGTTGCCTAACCATCTG | | Sma1 |
| HCRc-4 | Inserting Sma1 | | TCT AAGCTTTGTTGTAATATT | | HindIII |
| **Northern blotting** |  | |  | |  |
| T3 | Making probes for Northern blotting | | ATTAACCCTCACTAAAGGGA | |  |
| T7 | Making probes for Northern blotting | | TAATACGACTCACTATAGGG | |  |
| 18S rRNA N | 18S rRNA probe | | ACTTTCGATGGTAGGATAG | |  |
| 18S rRNA C |  |  | TGATCATCTTCGATCCCCTA | |  |
| GFP-start | GFP probe | | AAAACCGCGGATGTCTAAAGGTGAAGAATTATTCACTGG | | SacII |
| GFP-stop | GFP probe | | AAAGCGGCGCTTATTTGTACAATTCATCCATACCATGG | | NotI |
| Yst probe DNA XhoI F | QH probe | | GATCGCTCGAGATTGTTCATAATTAGCCATGTTGCACACC | | XhoI |
| Yst probe DNA XbaI R |  |  | GATCGTCTAGATTACAAGAGTTGCTATGTTAAAAAAGGCG | | XbaI |
| ALS3YN-XhoI-F | ALS3Y probe | | GATCGCTCGAGATGCAAATATTGGGGAGCAT | | XhoI |
| ALS3YN-XbaI-R |  |  | GATCGTCTAGAGGGGAAGCCTCTTTTATATACATTC | | XbaI |
| ALS3H-XhoI-F | ALS3H probe | | GATCGCTCGAGCCACTTTACAATCCCCATCTG | | XhoI |
| ALS3H-XbaI-R |  |  | GATCGTCTAGAGCGATTGAGATTGGTTGGTT | | XbaI |
